# Subtyping intractable functional constipation in children using clinical and laboratory data in a classification model

**DOI:** 10.3389/fped.2023.1148753

**Published:** 2023-04-24

**Authors:** Yi-Hsuan Huang, Chenjia Xie, Chih-Yi Chou, Yu Jin, Wei Li, Meng Wang, Yan Lu, Zhifeng Liu

**Affiliations:** ^1^ Department of Gastroenterology, Children’s Hospital of Nanjing Medical University, Nanjing, China; ^2^ Medical School, Nanjing University, Nanjing, China; ^3^ School of Electronic Science and Engineering, Nanjing University, Nanjing, China; ^4^ College of Medicine, National Taiwan University, Taipei, Taiwan; ^5^ Department of Quality Management, Children's Hospital of Nanjing Medical University, Nanjing, China

**Keywords:** intractable constipation, constipation subtypes, children, machine learning, quality of life

## Abstract

**Background:**

Children with intractable functional constipation (IFC) who are refractory to traditional pharmacological intervention develop severe symptoms that can persist even in adulthood, resulting in a substantial deterioration in their quality of life. In order to better manage IFC patients, efficient subtyping of IFC into its three subtypes, normal transit constipation (NTC), outlet obstruction constipation (OOC), and slow transit constipation (STC), at early stages is crucial. With advancements in technology, machine learning can classify IFC early through the use of validated questionnaires and the different serum concentrations of gastrointestinal motility-related hormones.

**Method:**

A hundred and one children with IFC and 50 controls were enrolled in this study. Three supervised machine-learning methods, support vector machine, random forest, and light gradient boosting machine (LGBM), were used to classify children with IFC into the three subtypes based on their symptom severity, self-efficacy, and quality of life which were quantified using certified questionnaires and their serum concentrations of the gastrointestinal hormones evaluated with enzyme-linked immunosorbent assay. The accuracy of machine learning subtyping was evaluated with respect to radiopaque markers.

**Results:**

Of 101 IFC patients, 37 had NTC, 49 had OOC, and 15 had STC. The variables significant for IFC subtype classification, according to SelectKBest, were stool frequency, the satisfaction domain of the Patient Assessment of Constipation Quality of Life questionnaire (PAC-QOL), the emotional self-efficacy for Functional Constipation questionnaire (SEFCQ), motilin serum concentration, and vasoactive intestinal peptide serum concentration. Among the three models, the LGBM model demonstrated an accuracy of 83.8%, a precision of 84.5%, a recall of 83.6%, a f1-score of 83.4%, and an area under the receiver operating characteristic curve (AUROC) of 0.89 in discriminating IFC subtypes.

**Conclusion:**

Using clinical characteristics measured by certified questionnaires and serum concentrations of the gastrointestinal hormones, machine learning can efficiently classify pediatric IFC into its three subtypes. Of the three models tested, the LGBM model is the most accurate model for the classification of IFC, with an accuracy of 83.8%, demonstrating that machine learning is an efficient tool for the management of IFC in children.

## Introduction

Functional constipation (FC) is estimated to affect 7.5% to 12.1% of children worldwide (1). The diagnosis of FC is based on symptoms and is defined according to the Rome IV diagnostic criteria (2, 3). Although the majority of children with FC respond well to conventional treatments, up to one-third of children with IFC respond less favorably (4). Intractable functional constipation (IFC) is defined as the inability of children to pass stool even with the maximum laxative treatment, daily rectal stimulation, behavioral therapy, or toilet training program for more than 3 months (4, 5).

IFC has a negative impact on the quality of life, affecting children and their families; they have poorer scores in physical, emotional, social, and school functioning domains compared to those of healthy populations (6). Children with constipation often present with symptoms like infrequent bowel movements, bloating, abdominal pain, and fecal incontinence (7). If not properly treated, a fourth of these children will continue to experience symptoms as adults (8). Thus, tools for accurately determining IFC subtype are highly essential for more effective management.

Colonic transit time (CTT), which is how long it takes for stool to pass through the colon, is used to classify IFC into three subtypes: normal transit constipation (NTC), outlet obstruction constipation (OOC), and slow transit constipation (STC) (9). The CTT of NTC patients is the same as the CTT for healthy individuals and NTC patients are able to expel stool without problems like healthy individuals do but they still report symptoms of constipation like bloating, abdominal pain, and hard stool (10, 11). OOC patients have a slightly longer than normal CTT due to impaired rectal contraction, paradoxical anal contraction, or inadequate anal relaxation; they are unable to expel stool after it reaches the rectum (12). STC patients have impaired colonic motility and contractility resulting in a prolonged CTT (13).

Because different subtypes have different managements it is important to determine the subtype as early as possible (14–16). For NTC patients, dietary fiber, osmotic laxatives, or prosecretory agents have limited effects. They should instead be referred for psychiatric consultations (4). OOC patients are less likely to respond to laxative therapy and rather need biofeedback therapy, which helps to train pelvic floor muscles involved in defecation (17). STC patients are treated with stimulants or colonic prokinetics and oftentimes require surgical management such as antegrade continence enemas, transcutaneous electrical stimulation, or colectomy (18).

Different pathophysiologic mechanisms have been proposed for different IFC subtypes (19). Numerous studies have demonstrated a substantial relationship between constipation and gastrointestinal hormones which are secreted by enteroendocrine cells dispersed throughout the gastrointestinal tract and act as crucial signaling molecules (20, 21). Some gastrointestinal hormones have been shown to affect colonic motility, including motilin (MTL), vasoactive intestinal peptide (VIP), ghrelin (GHRL), cholecystokinin (CCK) and glucagon-like peptide (GLP-1). MTL is widely distributed in the brain and gut and is known to stimulate gastrointestinal motility and regulate phase III of the migrating motor complex (MMC) (22, 23). VIP is an important inhibitory neurotransmitter in the enteric nervous system that relaxes the smooth muscle of the gastrointestinal tract (24). GHRL can regulate gut motility, adjust intestinal smooth muscle contraction, increase food intake, and secrete gastric acid (25). The motor effects of CCK include postprandial inhibition of gastric emptying and inhibition of colonic transit (26). The physiological role of GLP-1 is to balance energy, enhance glucose-stimulated insulin secretion, and reduce gastric emptying and gastrointestinal motility (27). In an adult study, the gastrointestinal hormone profiles were indicated as a reliable tool to classify IFC into the 3 subtypes; however there has not been a similar study conducted in children (28).

CTT evaluation using the radiopaque markers (ROM) test for subtyping IFC in children (29). This test requires children to swallow radiopaque marker capsules and takes at least 3 days to obtain and interpret the results (9). It is not only time-consuming but also exposes children to high doses of radiation (30). It can also be difficult for younger children to cooperate and swallow the ROMs. Alternative evaluation methods should be explored. Machine learning (ML) has been applied to a wide range of uses in healthcare such as solving diagnostic and prognostic problems (31). ML can identify complex patterns in multiple diverse data sources and increase prediction accuracy by using these uncovered patterns to classify disease phenotypes (32). However, few studies have applied ML to constipation (33, 34) and no study has used ML to analyze clinical and laboratory data to subtype IFC in children.

The aims of the present study are to (1) investigate the differences in clinical symptoms, quality of life, and self-efficacy through the use of constipation questionnaires in children with different subtypes of IFC; (2) evaluate their MTL, GHRL, VIP, CCK, and GLP-1 serum concentrations; (3) use a supervised machine learning model to subtype IFC in children.

## Materials and methods

### Study design

This was a single-center, cross-sectional study conducted at *Children's Hospital of Nanjing Medical University* (Nanjing, Jiangsu, China). The participants were recruited from the outpatient clinic of the Department of Gastroenterology. The study was conducted between December 2021 and July 2022. The researchers acquired the approval of the institutional review board (202110090-1) from the hospital where the first author worked and the site where participants were recruited.

### Participants

**Figure 1:**
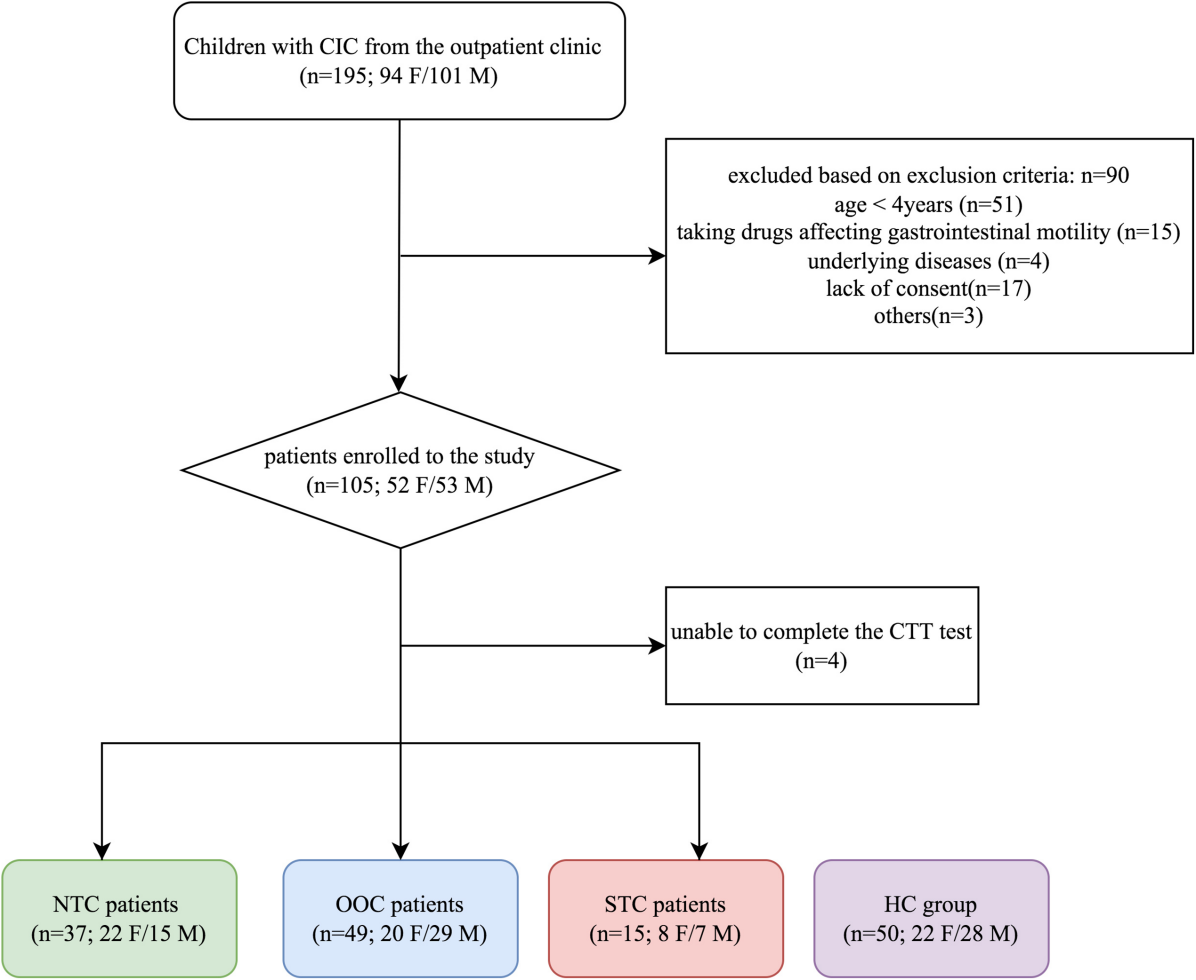
Flow chart of participants throughout the study. IFC, chronic intractable constipation; NTC, normal transit constipation; OOC, outlet obstruction constipation; STC, slow transit constipation; HC, healthy controls; F, female; M, male.

Participants were eligible for the study if they were between the ages of 4–14, had IFC (fulfilling the Rome IV criteria for FC), and responded poorly to 3 or more months of conventional therapy. Children with congenital abnormalities (i.e., cystic fibrosis, Hirschsprung disease, neuronal intestinal dysplasia, and anorectal malformations), anismus, constipation due to irritable bowel syndrome, inflammatory bowel disease, endocrine and metabolic diseases, diseases of myopathy and enteric nervous system, or a history of gastrointestinal surgery were excluded from the study. Patients taking any drug affecting gastrointestinal motility were excluded from the study (Figure 1).

### Data collection

During the preliminary visit, participants and their legal guardians were asked about the participant's medical history and were asked to complete the Chinese version of the Patient Assessment of Constipation Symptoms (PAC-SYM) (35), the Self-efficacy for FC questionnaire (SEFCQ) (36), and the Patient Assessment of Constipation Quality of Life (PAC-QOL) (37) for quantification of the participant's clinical symptoms and quality of life. Impartial assistance was provided to anyone who had difficulty understanding. Participants were given a physical examination which included an abdominal examination, an inspection of the perianal region, and an examination of the lumbosacral region. Venous blood samples were obtained for biochemical analysis.

During the second visit (participants were instructed to refrain from using laxatives or enemas 72 h prior to), the participants' CTT's were evaluated using radiopaque markers. The four children who failed to complete the CTT test were excluded from this study.

Children in the healthy control (HC) group were also given physical examinations and were also evaluated with the questionnaires of PAC-SYM, SEFCQ, and PAC-QOL. They were not evaluated for CTT because children without IFC lacked sufficient indications for this test.

### Patient assessment of constipation symptoms (PAC-SYM)

PAC-SYM is designed to assess the patient's experience of constipation in the last 2 weeks by quantifying symptoms and symptom severity. It is a self-reported questionnaire consisting of 12 symptoms divided into three domains: abdominal, rectal, and stool; the responses are rated on a 5-point Likert scale. The overall score is a sum ranging from 0 to 48, with higher values indicating more severe symptoms.

### Self-efficacy for FC questionnaire (SEFCQ)

SEFCQ is a disease-specific tool for evaluating self-efficacy in children with constipation and has been proven to be a good predictor of self-efficacy in terms of toilet training and treatment outcomes. The Chinese version of the SEFCQ includes 14 items that measure action and emotional self-efficacy. Participants rate each item on a Likert scale of 1 (never) to 4 (always). The total score is the sum of all the items and ranges from 14 to 56, with higher scores indicating greater self-efficacy.

### Patient assessment of constipation quality of life (PAC-QOL)

PAC-QOL is a questionnaire that specifically assesses the quality of life of patients diagnosed with constipation. It contains 28 items divided into 4 domains: 4 items in physical discomfort, 8 items in psychosocial discomfort, 11 items in worries and discomfort, and 5 items in treatment satisfaction. The subscale is a Likert scale ranging from 0 (absent) to 4 (very severe). Each domain's score is the sum of the items in that domain with higher scores meaning greater negative effects on quality of life.

### Radiopaque marker (ROM) test

In this study, the definitions of NTC, OOC, and STC are based on CTT. The ROM test is widely accessible, non-invasive, inexpensive, and the most common option for assessing CTT (38, 39). In a ROM test, the patient ingests a dissolving capsule containing 24 radiopaque markers and an abdominal radiographic image is taken after 72 h (40). The radiopaque markers are visible on the x-ray and are quantified based on the number and location of the markers remaining in the colon (14). If the number of retained markers is less than 5, indicating a CTT of <72 h, the patient would be classified as NTC. If the number of retained markers is more than 5, indicating a CTT value of >72 h, the patient would be classified as having abnormal transit constipation. Children with abnormal constipation can then be divided into two groups: the OOC group (>50% of the markers retained mainly in the rectosigmoid area and left colon area) and the STC group (retained markers are distributed throughout the whole colon area) (41).

### Serum concentration of gastrointestinal motility-related hormones

Venous blood samples were collected after overnight fasting into standard vacuum tubes. Serum samples for hormone concentrations analysis were stored at −80°C. Concentration quantification of serum MTL, GHRL, VIP, CCK, and GLP-1 were performed using an enzyme-linked immunosorbent assay kit by Cusabio.

### Statistical analysis

The children in the IFC group and the control group were matched according to age and gender. The Kolmogorov-Smirnov test was used and the data was distributed non-normally (the data was presented with a median and a 25th-75th interquartile range). The chi-square test was used to compare the gender distributions. Mann-Whitney U and Kruskal-Wallis H tests were used for comparing nonnormally distributed variables among the control and IFC subtype groups. For the analysis of the dataset, Spearman correlation analysis was used with the results presented as a heatmap. A univariate analysis was used to identify fifteen variables that contributed to the three IFC subtypes (each with *p*-values <0.05) and would be suitable for the establishment of ML models.

The significance level for the *p-*value was set at 0.05. The statistical analyses were carried out using the Statistical Package for Social Science (SPSS 24.0, IBM Corporation, Chicago, IL) and the GraphPad software (Prism 8.0, GraphPad Prism Software Inc., San Diego, CA).

### Machine-learning modeling

The resulting dataset, including clinical features and gastrointestinal motility-related hormones, was analyzed with the Python programming language (version 3.9.12), utilizing the Scikit-Learn library (version 1.1.0). For the development of a machine learning model, the children were randomly divided into a training set and a validation set with a 7:3 ratio. The training set was used to develop the ML models, which were then validated with the validation set. Three machine learning methods are utilized to develop prediction models: support vector machine (SVM), random forest (RF), and light gradient boosting machine (LGBM), with 5-fold cross validation for performance evaluation. K-fold cross validation was chosen for its ease of application in selecting the most appropriate model for a given predictive modeling problem and providing skill estimates with low bias. SelectKBest was used to determine the characteristic features. A grid search of the different parameters of each model was performed to determine the best parameters of each model and improve its performance. Accuracy, precision, recall, and f1-score were used as performance indicators. The results were depicted by a confusion matrix.

## Results

### Participant demographics

There were 105 participants enrolled in the study and 101 of the participants between the ages of 5–9 years old (median age of 6) completed the ROM test and questionnaires. Of 101 IFC patients, 37 were NTC [15 males, between the ages of 5–10 years old (median age of 7)], 49 were OOC [29 males, between the ages of 5–7 years old (median age of 5)] and 15 were STC [7 males, between the ages of 5–11 years old (median age of 6)]. There were 50 participants in the control group [28 males, between the ages of 5–9 years old (median age of 7)]. The BMI of the control group, NTC group, OOC group, and STC group were 16.41 (15.10–18.41), 15.63 (14.61–16.59), 15.88 (14.82–17.00), and 16.53 (13.78–17.60), respectively. The Z-score of the control group, NTC group, OOC group, and STC group were 0.045 (−0.415–1.1), 0.03 (−0.5–0.47), −0.12 (−0.785–1.39), and −0.23 (−2.37–0.69) respectively. The BMI of the control group was generally within the normal BMI range (z-score: −1.0 to +1.0), leaning towards slightly overweight. The time taken per defecation attempt for the control group, NTC, OOC, and STC groups was 10 min (10–15), 10 min (10–20), 15 min (10–25), and 10 min (10–15) respectively. The stool frequency per week were 5 times/week (4–6), 2 times/week (1–2), 2 times/week (1–2) and 1 time/week (1–2), respectively. The Bristol stool consistency were type 4 (3– 4), type 2 (2–3), type 2 (1–2) and type 2 (1–2) respectively. Among IFC groups, the time duration of constipation was 3 years (2–5), 3 years (1.75–4), and 4 years (3–7) for NTC, OOC, and STC groups respectively. Overall, no significant differences were found among the gender, ages, BMIs, and Z-scores of the 4 groups. There were significant differences in stool frequency and Bristol stool scale between the HC and IFC subtype groups (*p *< 0.001), with no significant difference within each subtype (all *p* > 0.05). The participant demographics are shown in Table 1.

**Table 1:** Participant demographics.

Items	HC	NTC	OOC	STC	*p*-value
Participants (*n*)	50	37	49	15	
Males (*n*)	28 (56%)	15 (40.5%)	29 (59.2%)	7 (46.7%)	0.328
Females (*n*)	22 (44%)	22 (59.5%)	20 (40.8%)	8 (53.3%)	
Age (year)	7 (5–9)	7 (5–10)	5 (5–7)	6 (5–11)	0.115
BMI (kg/m^2^)	16.41 (15.10–18.41)	15.63 (14.61–16.59)	15.88 (14.82–17.00)	16.53 (13.78–17.60)	0.272
Z-score	0.045 (−0.415–1.1)	0.03 (−0.5–0.47)	−0.12 (−0.785–1.39)	−0.23 (−2.37–0.69)	0.464
Duration of constipation (year)	–	3 (2–5)	3 (1.75–4)	4 (3–7)	0.186
Time per attempt (minute)	10 (10–15)	10 (10–20)	15 (10–25)	10 (1–15)	0.883
Stool frequency (week)	5 (4–6)	2 (1–2)	2 (1–2)	1 (1–2)	<0.001
Bristol stool scale	4 (3–4)	2 (2–3)	2 (1–2)	2 (1–2)	<0.001

Data expressed as median (interquartile range).

Binomial data expressed in percentage distribution.

NTC, normal transit constipation; OOC, outlet obstruction constipation; STC, slow transit constipation; BMI, Body Mass Index.

### Clinical characteristics

We compared the PAC-SYM, PAC-QOL, and SEFCQ scores among the four groups. Table 2 shows the medians of the PAC-SYM, PAC-QOL, and SEFCQ scores of the NTC, OOC, STC, and HC groups. There were significant differences in PAC-SYM scores among the four groups (*p *< 0.001). Pairwise comparison between the HC group and IFC subtype groups also showed significant differences (*p *< 0.001); however, the NTC, OOC, and STC groups presented similar scores in all the PAC-SYM subscales (abdominal: *p* = 0.631; rectal: *p* = 0.762; stool: *p* = 0.686). There were significant differences (*p *< 0.001) in SEFCQ scores among the four groups. Pairwise comparison between the HC group and IFC subtype groups significant differences (*p *< 0.001). Significant differences were also found in PAC-QOL scores among the four groups (*p *< 0.001). Pairwise comparison between the HC group and IFC subtype groups showed significant differences (*p *< 0.001). NTC, OOC, and STC groups showed similar PAC-QOL scores in the 3 domains: worries and concerns, psychosocial discomfort, and physical discomfort (*p *= 0.853, *p *= 0.953, and *p *= 0.347 respectively). However, there was a significant difference in the satisfaction domain among the three subtypes (Kruskal-Wallis Test: *p *= 0.018) for the PAC-QOL subscale score of the satisfaction among the 3 groups. The comparisons of the NTC, OOC, and STC groups are shown in Supplementary Table S1.

**Table 2:** Clinical characteristics.

	HC	NTC	OOC	STC	*p*-value
PAC-SYM (Abdominal)	1 (0–1)	2 (0–4)	2 (0–4)	3 (2–4)	<0.001
PAC-SYM (Rectal)	0 (0–1)	6 (3–8)	7 (5–8)	7 (3–8)	<0.001
PAC-SYM (Stool)	0 (0–0)	14 (10–17)	15 (11–19)	15 (9–19)	<0.001
SEFCQ (Action)	27 (27–28)	23 (20–26)	24 (21–27)	13 (13–17)	<0.001
SEFCQ (Emotion)	28 (27–28)	27 (23–28)	21 (18–24)	13 (13–17)	<0.001
PAC-QOL (Worries and concerns)	1 (0–1)	16 (6–21)	19 (8–22)	15 (12–21)	<0.001
PAC-QOL (Psychosocial discomfort)	0 (0–1)	9 (6–13)	8 (6–12)	8 (3–14)	<0.001
PAC-QOL (Physical discomfort)	0 (0–1)	3 (1–5)	4 (1.5–5)	5 (2–7)	<0.001
PAC-QOL (Satisfaction)	0 (0–0)	15 (11–17)	16 (13–17)	17 (16–18)	<0.001

Data expressed as median (interquartile range).

PAC-SYM, the Patient Assessment of Constipation Symptoms; SEFCQ, the Self-efficacy for FC questionnaire; PAC-QOL, the self-reported Patient Assessment of Constipation Quality of Life; HC, healthy control; NTC, normal transit constipation; OOC, outlet obstruction constipation; STC, slow transit constipation.

### Serum concentration of gastrointestinal motility-related hormones

Table 3 shows the serum concentrations of gastrointestinal motility-related hormones (MTL, GHRL, VIP, CCK, and GLP-1) of the NTC, OOC, STC, and HC groups. NTC, OOC, and STC patients had less MTL compared to the patients in the HC group (*p *< 0.001) and STC patients showed the most reduced MTL level among all groups (STC vs. NTC, *p *< 0.001; STC vs. OOC, *p *< 0.001; NTC vs. OOC, *p *= 0.437). VIP release was significantly higher in STC patients compared to NTC, OOC, and HC patients (STC vs. NTC, *p *< 0.001; STC vs. OOC, *p *< 0.001; NTC vs. OOC, *p *= 0.570). In addition, the HC group's VIP was significantly lower than that of all the IFC subtypes (*p *< 0.001). Serum CCK and GHRL concentrations were lower in the IFC groups than in the HC group (*p *< 0.001), though these two hormones did not differ statistically among the NTC, OOC, and STC groups. On the other hand, unlike the other gastrointestinal motility-related hormones, the concentrations of GLP-1 were similar between IFC patients and healthy controls. The pairwise comparisons of the HC, NTC, OOC, and STC groups are shown in Supplementary Table S2.

**Table 3:** Serum concentration of gastrointestinal motility-related hormones.

	HC	NTC	OOC	STC	*p*-value
MTL (pg/ml)	57.50 (49.04–69.53)	29.28 (21.59–44.49)	28.18 (23.05–35.59)	15.31 (13.61–18.64)	<0.001
VIP (pg/ml)	19.38 (16.18–26.93)	56.27 (32.12–68.24)	49.09 (29.93–63.96)	88.21 (72.00–92.78)	<0.001
GHRL (pg/ml)	8,689 (7,961–8,946)	7,618 (6,445–8,406)	7,561 (6,617–8,607)	7,967 (7,237–8,291)	<0.001
CCK (pg/ml)	307.3 (283.9–346.1)	209.8 (133.0–294.1)	191.0 (133.0– 289.7)	183.3 (127.7–237.0)	<0.001
GLP-1 (ng/ml)	1.950 (1.517–2.890)	1.960 (1.630–2.900)	1.920 (1.505–2.630)	2.610 (1.440–3.310)	0.869

Data expressed as median (interquartile range).

MTL, motilin; VIP, vasoactive intestinal peptide; GHRL, ghrelin; CCK, cholecystokinin; GLP-1, glucagon-like peptide; HC, healthy control; NTC, normal transit constipation; OOC, outlet obstruction constipation; STC, slow transit constipation.

### Machine-learning analysis

**Figure 2:**
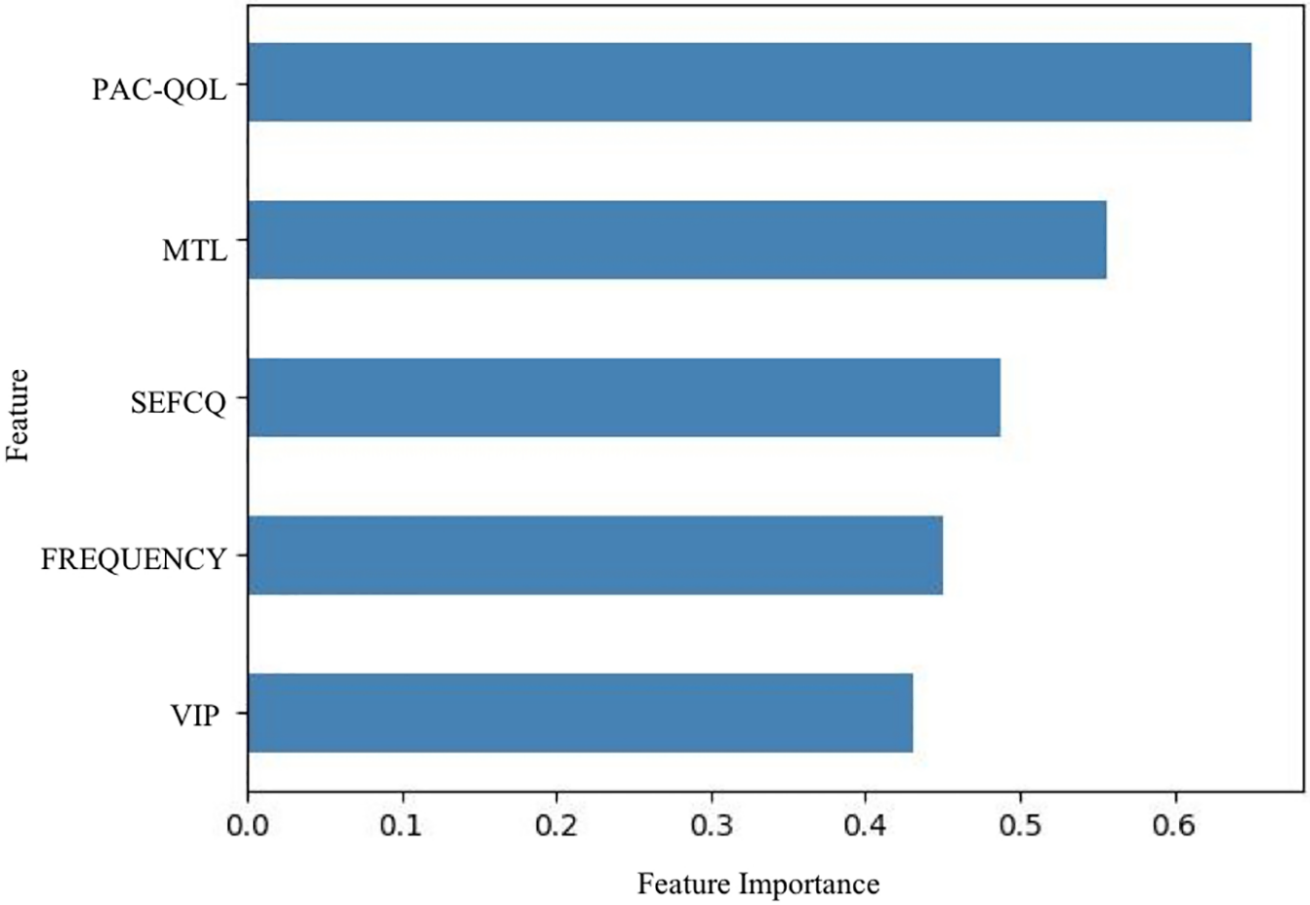
Feature importance of variables estimated by selectKBest. The importance of the features were evaluated by SelectKBest. Of the 15 features that were evaluated, these are the top 5 features used in the prediction models, stool frequency, PAC-QOL, SEFCQ, MTL, VIP. FREQUENCY, stool frequency; PAC-QOL, PAC-QOL (Satisfaction); SEFCQ, SEFCQ (Emotion); MTL, motilin; VIP, vasoactive intestinal peptide.

**Figure 3:**
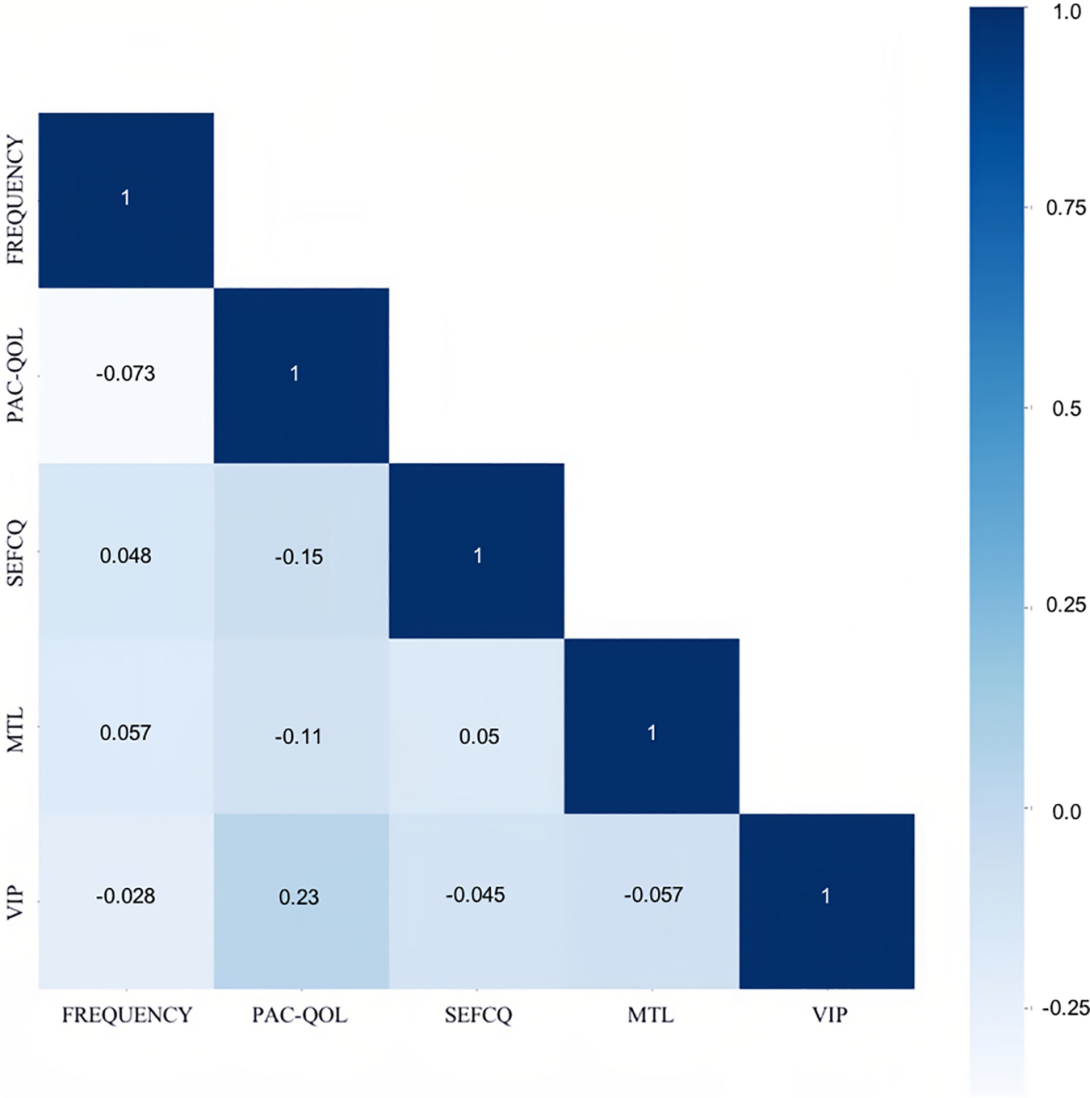
The correlation of the best variables through the heat map. The Spearman correlation was used for this heat map matrix of the top 5 variables as identified by SelectKBest. Each variable on the heat map has a correlation coefficient represented by the color gradient scale. The more positively correlated the features are the darker the shades of blue. FREQUENCY, stool frequency; PAC-QOL, PAC-QOL (Satisfaction); SEFCQ, SEFCQ (Emotion); MTL, motilin; VIP, vasoactive intestinal peptide.

**Figure 4:**
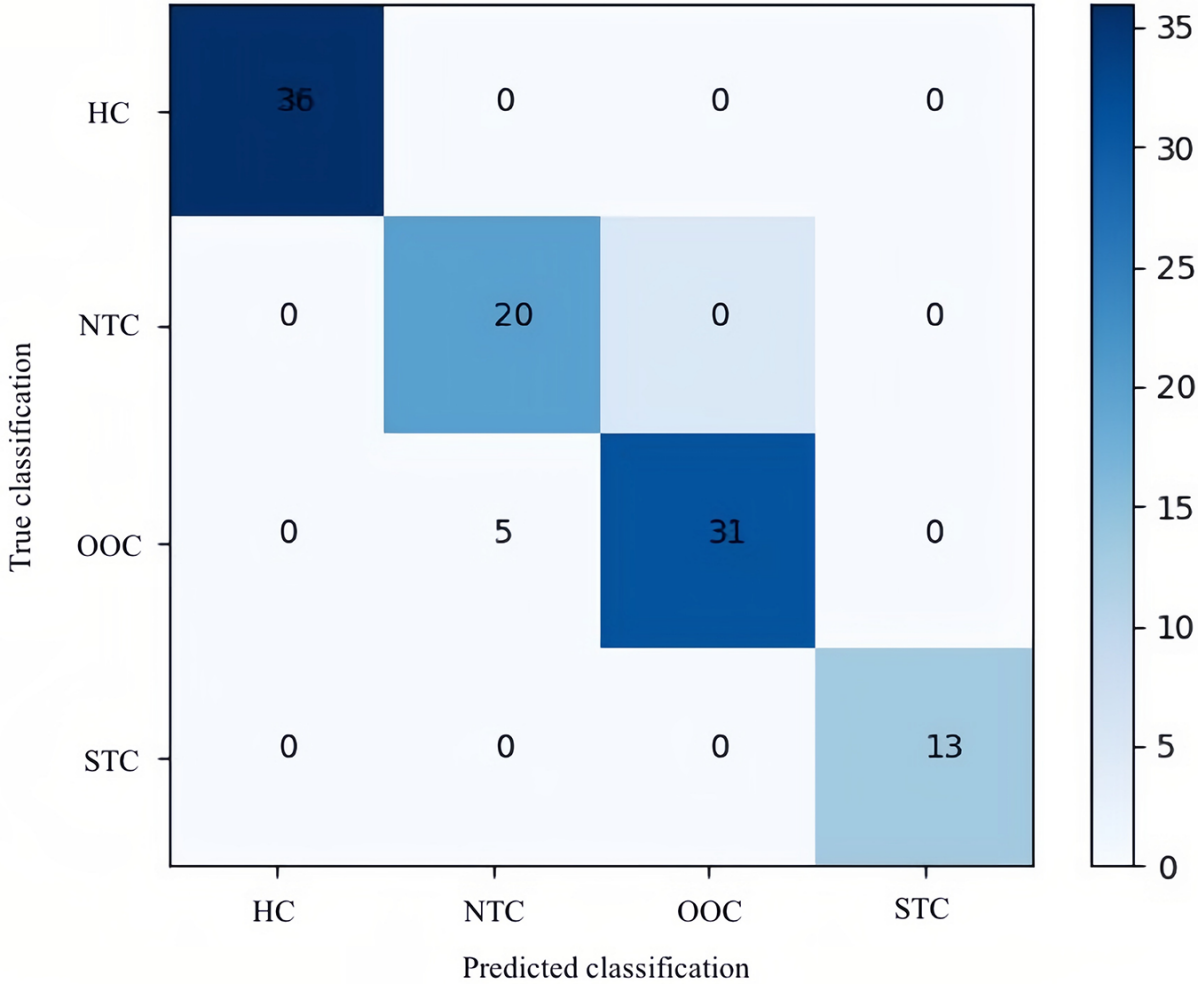
Confusion matrix of the optimal LGBM model. This confusion matrix depicts the accuracy of the LGBM model in IFC subtype classification. The horizontal axis is the predicted label of IFC subtype classification, and the vertical axis is the true label. According to the matrix, of the 36 healthy controls included in the test data, 36 were predicted correctly; of the 25 children with NTC, 20 were predicted correctly; of the 31 children with OOC all were predicted correctly; and of the 13 children with STC, 13 all were predicted correctly by LGBM. HC, healthy controls; NTC, normal transit constipation; OOC, outlet obstruction constipation; STC, slow transit constipation.

**Figure 5:**
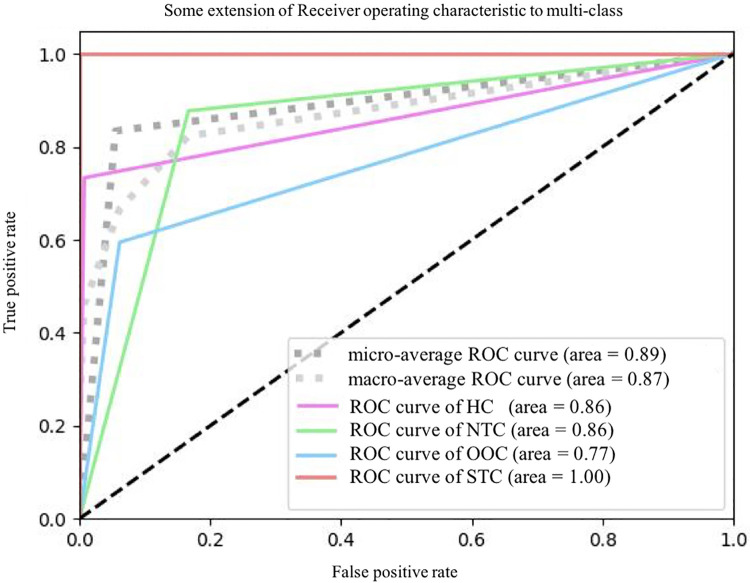
The ROC plots of LGBM classification. ROC, receiver operating characteristic curve; HC, healthy controls; NTC, normal transit constipation; OOC, outlet obstruction constipation; STC, slow transit constipation.

In terms of significant variables, stool frequency, PAC-QOL (Satisfaction), SEFCQ (Emotion), MTL, and VIP were chosen by SelectKBest. Figure 2 shows the feature importance of the variables while Figure 3 is a heat map that illustrates the correlation of the 5 variables. We developed three ML algorithms using SVM, RF, and LGBM. These algorithms were trained using the 5 features from the same dataset. We then validated our three ML algorithms using a stratified 5-fold cross validation. Among the three ML models, SVM and RF showed an accuracy of 79.0% and 80.0%, respectively. The optimized LGBM model outperformed the other two with an 83.8% accuracy. The SVM model had a precision of 82.7%, a recall of 81.7%, and a f1-score of 80.3%. The RF model had a precision of 80.4%, a recall of 79.6%, and a f1-score of 79.6%. The LGBM model had a precision of 84.5%, a recall of 83.6%, and a f1-score of 83.4% (Table 4). Figure 4 shows the confusion matrix demonstrating the results of the LGBM model prediction vs. CTT results. The LGBM model was able to accurately classify all those in the HC group, correctly subtype all those in the NTC and STC groups, and manage to correctly subtype 31 out of 36 of those in the OOC group (5 were misclassified as NTC patients). Figure 5 shows the receiver operating characteristic curves for IFC subtype classification of the LGBM model.

**Table 4:** Predictive performance of the three models in classifying IFC subtype in children.

Models	Accuracy (%)	Precision (%)	Recall (%)	F1-score (%)
**Training Set**				
SVM	79.0	82.7	81.7	80.3
RF	80.0	80.4	79.6	79.6
LGBM	83.8	84.5	83.6	83.4
**Validation Set**				
SVM	87.6	89.6	85.5	86.6
RF	95.2	95.5	95.0	95.1
LGBM	95.2	96.5	95.0	95.1

SVM, support vector machine; RF, random forest; LGBM, light gradient boosting machine.

## Discussion

Children without alarming symptoms of functional constipation, including signs of congenital abnormalities, stunted growth, and symptoms suggestive of sexual abuse, are not routinely recommended to undergo early diagnostic screening (4). However, it is estimated that nearly half of the children with FC do not recover and still depend on laxatives even after 6 to 12 months of treatments (42, 43). Some of these children have poor responses to conservative treatment strategies, with persistent severe symptoms impacting their quality of life (6). Early diagnosis of IFC subtype enables clinicians to find better management strategies and more effective treatments for IFC children rather than using one generalized treatment method. This study was designed to identify the differences in clinical characteristics using specific constipation questionnaires and gastrointestinal motility-related hormones of children with IFC subtypes. Among the variables of the dataset, stool frequency, PAC-QOL (Satisfaction), SEFCQ (Emotion), MTL, VIP are the most distinguishing features of IFC with significant differences between subtypes. To the best of our knowledge, this is the first study that uses ML algorithms to subtype IFC in children. Out of the three ML models investigated, LGBM performed the best, with an accuracy of 83.8%.

One of our aims was to investigate the clinical differences among the HC, NTC, OOC, and STC groups in the pediatric population. We evaluated constipation symptoms, quality of life, and defecation self-efficacy through the use of specific questionnaires (e.g., PAC-SYM, SEFCQ, and PAC-QOL) as well as clinical tools like the Bristol stool scale. Stool frequency, SEFCQ (Emotion), and PAC-QOL (Satisfaction) were selected for model development. Riezzo et al. observed that the frequency of defecation between STC and NTC groups showed a significant difference (28). Jiang et al. reported that OOC patients had more severe constipation symptoms and significantly worse PAC-SYM (Stool) scores than STC and NTC patients in the Chinese chronic constipation adult population (44). Santucci et al. stated that the SEFCQ (Emotion) scale addresses emotions associated with a child's ability to defecate like worry and sadness, which relate to the fear of painful defecation. Low defecation self-efficacy can cause anxiety and may contribute to decreased compliance of behavioral change, which exacerbates constipation symptoms (36). Our PAC-QOL results of the STC group are consistent with those of Melanie et al. who pointed out that children with STC may experience impaired physical and emotional functions (45). The Bristol stool scale ranks stool consistency from type 1 (hard separate lumps) to type 7 (liquid with no solid pieces) and is widely used in clinical practice and research (46). Saad et al. discovered that types 1 and 2 of the Bristol stool scale were predictive of STC in 46 patients with IFC (47).

The pathogenesis of FC is still yet unknown and though some studies have noted the possible role of gastrointestinal motility-related hormones. Thus, the second aim of this study was to assess the serum levels of MTL, GHRL, VIP, CCK, and GLP-1 in children with NTC, OOC, and STC in comparison to those of healthy controls. MTL and VIP were selected, out of all the serum hormone concentrations we evaluated, as two of the features used in our models. In our study, there was a considerable difference in MTL serum levels between the HC group and the 3 IFC subtype groups. Moreover, the MTL serum level of the STC group was lower than that of the OOC and NTC groups. Our findings are consistent with previous research investigating FC in patients with decreased MTL levels in the fasting state (48). Peracchi et al. showed that MTL decreased in the postprandial state in STC patients (49). Additionally, Giuseppe Riezzo et al. demonstrated that MTL level was lower in the STC group than in the control and NTC groups (28). It has also been observed that children with constipation have lower serum MTL levels than healthy children do (50). In our study, the serum VIP levels of IFC subtype groups were significantly increased compared to those in the HC group. In addition, the serum levels of STC groups were much higher than those of the OOC and NTC groups. VIP has been identified as an inhibitory neurotransmitter that relaxes the smooth muscles of the gastrointestinal tract, thereby restraining gastrointestinal motility (51). The levels of neurotransmitters are closely related to the motility of the gastrointestinal tract (34, 52, 53). An *in vivo* experiment in rats by Zhu et al. has found that an excessively high level of VIP causes intestinal peristalsis to slow down inducing constipation; the serum VIP level decreased after treatment and symptoms were relieved (54). A clinical study by Ling Cheng et al. found that the colon muscle cells of female STC patients have an increased response to inhibitors like VIP that stimulate G protein-coupled receptors (55). Zhizhuwan, a traditional Chinese medicine regulating GI motility, greatly relieved constipation symptoms and decreased VIP level in patients with constipating diabetes mellitus (56).

At present, the only way to subtype IFC in children is with the ROM test to determine the child's CTT (9). However, it is difficult for children to swallow the radiopaque marker capsules and it takes at least 3 days to obtain and interpret the results. Machine learning (ML) can rapidly process high-dimensional data and identify correlations between many variables, making it very efficient when used for disease prediction, diagnosis, and precision (57, 58). Logistic regression, SVM, K Nearest Neighbors, Decision Trees, RF, and Gradient Boosting Machine have been frequently used in pediatric studies (59–62). Specifically, applications of supervised machine learning have been used in studies for disease prediction (63). The majority of artificial intelligence research in gastroenterology focuses on adult diseases but a number of pediatric diseases could benefit from more studies in ML fields (64). Jasbir Dhaliwal et al. used a similarity network fusion and RF in a retrospective study to subtype pediatric inflammatory bowel disease with an accuracy of 97% (65). In a prospective study, De Meij et al. used a logistic ridge regression model to analyze microbiota profiles and discriminate 61 healthy controls from 76 children with chronic functional constipation with an accuracy of 82% (33). With fundamental developments in handling complex and multidimensional datasets, traditional statistical methods used in the past are now being gradually replaced by machine learning-based clinical decision support systems (66).

Herein, we analyzed the dataset including clinical characteristics and gastrointestinal motility-related hormones using ML algorithms to classify patients with IFC. SelectKBest data analysis was used for feature selection. For this study, three models used, SVM and RF are considered more conventional models while LGBM is a more recent model that is being utilized in classification of diseases. To avoid overfitting, k-fold cross validation was used. A forward selection was performed with these three models to optimize the model performance. The LGBM model showed the best performance for classification in our case. Tree-based ML models are known to show good results for classification (67–69). Tsai et al. reported that the LGBM model had a superior performance in discriminating patients with bladder cancer from patients with cystitis based on clinical laboratory data, showing its potential as a faster and more cost-effective diagnostic tool (70). Rudo et al. demonstrated that the LGBM model outperformed KNN, SVM, NB, Bagging, RF, and XGBoost in the prediction of diabetes mellitus in countries with a low concentration of medical experts as a possible means of improving healthcare quality with fewer physicians (71). Peng et al. found that LGBM performed better than other clinical models in early identification of acute kidney injury in congestive heart failure patients (72). This model also helped in treatment planning, determining whether renal replacement therapy was need as well as in assessing the short-term prognosis of the patients. The developed LGBM model is deemed to be very effective for supporting physicians in the early management of a variety of diseases including intractable constipation in children.

The limitations of this study should be noted. We could not compare CTT among all groups and evaluate any possible correlation between CTT and gastrointestinal motility-related hormones for all participants because there were insufficient indications to perform the ROM test on healthy children. Also, we did not analyze the serum levels before and after treatment or the changes in stool consistency and symptom relief. Because all participants of this case-control study were recruited from the children's hospital, hospital control bias was inevitable, though we did our best to mitigate this by recruiting participants with a variety of conditions. For the ML analysis, because the sample size of this single-center design is relatively small, external validation was not available. Further verification through prospective clinical studies with a significantly larger prospective cohort of patients is required to confirm the findings of this study.

## Conclusion

Children with IFC suffering from severe symptoms (i.e., infrequent defecation, painful bowel movements, and abdominal pain) and responding poorly to the standard conservative treatment will often continue to experience these symptoms in adulthood. The persistence of IFC has a negative impact on their growth, development, and quality of life. With early subtyping of IFC, clinicians can provide management options that target the cause of their constipation whether it is psychiatric therapy for NTC patients, biofeedback therapy to train pelvic muscles for OOC patients, or laxatives and surgery for STC patients. The current method of subtyping IFC using the ROM test to evaluate CTT is time-consuming and involves radiation exposure. Younger children also have more difficulties cooperating with the ROM test. Other methods of subtyping should be considered and further evaluated for more efficient diagnosis of IFC subtypes. Our study indicated the potential of applying ML models to analyze clinical data and gastrointestinal hormone profiles to subtype IFC. This study appears to be the first that uses the LGBM model to subtype IFC in children, shedding new light on the values of features that were not previously recognized.

## Data Availability

The original contributions presented in the study are included in the article/**Supplementary Material**, further inquiries can be directed to the corresponding author/s.
