# Supplementary material for: Subtyping intractable functional constipation in children using clinical and laboratory data in a classification model
Source: Front Pediatr. 2023 Apr 24;11:1148753. doi: 10.3389/fped.2023.1148753 (PMC10165123; doi:10.3389/fped.2023.1148753)
Supplement: Supplementary file 1 [file Table1.docx]

Table S1. Comparisons of Clinical Characteristics in NTC, OOC, and STC groups

|  | NTC (n=37) | OOC (n=49) | STC (n=15) | *p*-value |
| --- | --- | --- | --- | --- |
| PAC-SYM (Abdominal) | 2(0–4) | 2(0–4) | 3(2–4) | 0.631 |
| PAC-SYM (Rectal) | 6(3–8) | 7(5–8) | 7(3–8) | 0.762 |
| PAC-SYM (Stool) | 14(10–17) | 15(11–19) | 15(9–19) | 0.686 |
| SEFCQ (Action) | 23(20–26) | 24(21–27) | 13(13–17) | ＜0.001 |
| SEFCQ (Emotion) | 27(23–28) | 21(18–24) | 13(13–17) | ＜0.001 |
| PAC-QOL (Worries and concerns) | 16(6–21) | 19(8–22) | 15(12–21) | 0.853 |
| PAC-QOL (Psychosocial discomfort) | 9(6–13) | 8(6–12) | 8(3–14) | 0.953 |
| PAC-QOL (Physical discomfort) | 3(1–5) | 4(1.5–5) | 5(2–7) | 0.347 |
| PAC-QOL (Satisfaction) | 15(11–17) | 16(13–17) | 17(16–18) | 0.018 |

SEFCQ (Action): STC versus NTC, p=0.002; STC versus OOC, p<0.001; SEFCQ (Emotion): STC versus OOC, p=0.049; NTC versus STC, p<0.001; OOC versus NTC, p<0.001.; PAC-QOL (Satisfaction): NTC versus STC, p=0.027.

PAC-SYM, the Patient Assessment of Constipation Symptoms; SEFCQ, the Self-efficacy for FC questionnaire; PAC-QOL, the self-reported Patient Assessment of Constipation Quality of Life; HC, healthy control; NTC, normal transit constipation; OOC, outlet obstruction constipation; STC, slow transit constipation.
