# Supplementary material for: Subtyping intractable functional constipation in children using clinical and laboratory data in a classification model
Source: Front Pediatr. 2023 Apr 24;11:1148753. doi: 10.3389/fped.2023.1148753 (PMC10165123; doi:10.3389/fped.2023.1148753)
Supplement: Supplementary file 2 [file Table2.docx]

Table S2. Pairwise Comparisons of Gastrointestinal Motility-related Hormones in NTC, OOC, and STC groups

|  | HC-NTC | HC-OOC | HC-STC | NTC-OOC | NTC-STC | OOC-STC |
| --- | --- | --- | --- | --- | --- | --- |
| MTL | <0.001 | <0.001 | <0.001 | 0.437 | <0.001 | <0.001 |
| VIP | <0.001 | <0.001 | <0.001 | 0.57 | <0.001 | <0.001 |
| GHRL | 0.005 | <0.001 | <0.001 | 0.451 | 0.503 | 0.891 |
| CCK | <0.001 | <0.001 | <0.001 | 0.533 | 0.557 | 0.285 |
| GLP-1 | – | – | – | – | – | – |

MTL, motilin; VIP, vasoactive intestinal peptide; GHRL, ghrelin; CCK, cholecystokinin; GLP-1, glucagon-like peptide; HC, healthy control; NTC, normal transit constipation; OOC, outlet obstruction constipation; STC, slow transit constipation.
